# Histone H3 activates caspase-1 and promotes proliferation and metastasis in hepatocellular carcinoma

**DOI:** 10.7150/ijms.76580

**Published:** 2023-04-09

**Authors:** Mengjia Jing, Lamu Qiong, Zi Wang, Xiaofeng Xiong, Yu Fu, Wei Yan

**Affiliations:** 1 Institute of Liver and Gastroenterology Diseases, Tongji Hospital, Tongji Medical College, Huazhong University of Science and Technology, Wuhan, Hubei, China.; 2 Department of Gastroenterology, the First Affiliated Hospital of Nanjing Medical University, Nanjing, Jiangsu, China.; 3 Department of Gastroenterology, Union Hospital, Tongji Medical College, Huazhong University of Science and Technology, Wuhan, Hubei, China.

**Keywords:** Histone H3, TLR9, NLRP3, Hypoxia, Hepatocellular carcinoma

## Abstract

**Background:** As a component of nucleosomes, histone H3 plays an important role in chromosome structure and gene expression. Current studies have mostly focused on the role of histones in epigenetics, but in addition to this, the role of histones themselves in tumor development and microenvironment have been less explored.

**Methods:** Western blot and immunofluorescence were carried out to detect the content and localization of histone H3 in hepatocellular carcinoma. The changes of histone H3 were observed in hypoxia treatment cells, the specific action mechanism of histone H3 was studied by CoIP and other methods. Cell Counting Kit-8 assay, plate cloning assay and transwell assay were used to exam the effect of histone H3 on cell proliferation and metastasis, which were verified by subcutaneous tumors in mice and lung metastasis by tail vein injection in mice.

**Results:** We found that histone H3 was overexpressed in hepatocellular carcinoma tumor tissues compared to adjacent non-tumor tissues, and there was concomitant translocation of histone H3 from the nucleus to the cytoplasm. We found that hypoxia could contribute to this phenomenon of histone H3 translocation from the nucleus to the cytoplasm in hepatocellular carcinoma cells and increased binding levels to TLR9. At the same time, hypoxia induced downstream activation of TLR9 and caspase-1, as well as cleavage and release of the pro-inflammatory cytokines IL-1β and IL-18. We further demonstrated that histone H3 could also promote proliferation and metastasis of hepatocellular carcinoma through TLR9 activation of NLRP3 inflammasome. In addition, overexpression of histone H3 was also confirmed to promote hepatocellular carcinoma proliferation and metastasis in mouse models of hepatocellular carcinoma growth assay and lung metastasis.

**Conclusions:** In hypoxic hepatocellular carcinoma cells, histone H3 can translocate to the cytoplasm and activate caspase-1 via TLR9, thereby producing pro-inflammatory cytokines that promote tumor proliferation and metastasis.

## Introduction

HCC (hepatocellular carcinoma) is one of the most lethal malignancies in the world due to its susceptibility to early metastasis and the lack of effective clinical treatment options^1^. However, HCC has a complex pathogenesis with great molecular heterogeneity. Therefore, the increasing research on the underlying mechanisms of HCC has become a focus of increasing attention for cancer researchers.

Hypoxia and chronic inflammation have long been recognized as important hallmarks of cancer cells and their microenvironment^2^. Under hypoxic conditions, cells undergo genetic and adaptive changes that allow them to survive and even proliferate and metastasize^3^. Inflammation can also contribute to the development and progression of cancer through multiple pathways, including DNA mutations caused by increased ROS (reactive oxygen species) and cell-mediated immune tolerance or failure^4^. Inflammasomes, signaling complexes composed of multiple intracellular proteins, are key signaling pathways for inflammation, and dysregulation of their activation has been linked to tumor pathogenesis^5^, and the clinical relevance of inflammasomes in multiple cancers highlights their therapeutic promise as molecular targets^6^. Among them, NLRP3, as a member of the most sought-after inflammasomes, is a "star" component of inflammation and can promote the development of various tumors, including breast cancer.

As an important component of nucleosomes, the basic structural unit of chromatin, histones not only maintain the structure of chromatin, but also play an important regulatory role in the transcriptional expression of genes^7^. It has been found that histones can contribute to tumor development in various ways, including post-translational modification of histones^8^, transcriptional regulation of histone variants, and deposition^9^. However, current studies have mostly focused on the role of histones in epigenetics, but in addition to this, new studies have found that histones may be released into the extracellular space that serve to stimulate inflammation development^9^.

The development of HCC is dependent on its local microenvironment, and hypoxia and inflammation are two key factors in the formation of the HCC microenvironment^11^. Our previous study found that HCC cells exposed to hypoxia showed no significant difference in cell viability compared with those exposed to normoxia, but their migration and metastatic abilities were significantly increased^12^. Further studies revealed that hypoxic HCC cells could induce activation of inflammasomes through extracellular release of HMGB1 (high mobility group protein 1) and activation of TLR4 (toll-like receptor 4) and RAGE (receptor for advanced glycation endproducts) signaling pathways. And one study found that endogenous histones caused inflammation through pattern recognition receptor TLR9 in an animal model of hepatic ischemia-reperfusion injury^13^. We tried to investigate the role of histone H3 on HCC cells in the hypoxic tumor microenvironment and the specific mechanism to provide new ideas for basic research and clinical treatment of HCC.

Therefore, we assessed whether histone H3 could induce caspase-1 activation and subsequently promote proliferation, metastasis and invasion of HCC cells. Our results show that histone H3 is overexpressed by tumors in certain HCC clinical specimens compared to adjacent non-tumor tissues. Similar to the phenomenon in ischemia-reperfusion liver injury^13^, histone H3 undergoes this change of translocation from the cytosol to the cytoplasmic and extracellular spaces during hypoxia, while the amount of histone H3 bound to TLR9 increases after hypoxia. Overexpression of histone H3 in HCC cells enhancs proliferation and lung metastasis in the mouse HCC models. In addition, TLR9 plays an important mediating role in the induction of caspase-1 activation by histone H3. Inhibition of TLR9 in stable histone H3 overexpressing cells reduces cell proliferation, metastasis, and invasiveness. This shows that in hypoxic HCC cells, histone H3 activates TLR9, induces caspase-1 activation and subsequently produces a variety of inflammatory mediators, which in turn promotes tumor proliferation and metastasis.

## Methods

### Patients and samples

All patient samples used in the study were obtained and stored at the Institute of Liver and Gastroenterology Diseases, Tongji Hospital, Tongji Medical College, Huazhong University of Science and Technology, and informed consent was obtained from all patients. These patients were all adult patients undergoing radical treatment in Tongji Hospital, and had pathological diagnosis of HCC. Some of these tissues were stored in liquid nitrogen tanks for immunoblot analysis, and some were fixed with 4% paraformaldehyde and embedded in paraffin for immunohistochemical or immunofluorescence staining analysis. The study was conducted in compliance with the requirements of the Ethics Committee of Tongji Hospital and in accordance with the ethical standards of the Declaration of Helsinki of the World Medical Association.

### Cell culture

Cells were incubated in DMEM (dulbecco's modified eagle medium) containing 10% FBS (fetal bovine serum) at 37°C in a humidified incubator with 5% CO_2_. When incubated under hypoxic conditions, the cells were transferred to an incubator containing 1% O_2_. Human hepatoma cell lines G2, Huh7, MHCC-97H were obtained from the Institute of Liver and Gastroenterology Diseases, Tongji Hospital, Tongji Medical College, Huazhong University of Science and Technology.

### Animals

BALB/c nude mice (male, 4 weeks old) were purchased from HFK Bioscience Ltd (Beijing, China) and bred under pathogen-free conditions. Animal experiments were approved by the Institutional Review Board of Tongji Hospital (ACUC No. 2378).

### Immunohistochemistry

The histochemical immunoassay kit was purchased from Wuhan Dingguo Biological Technology Co., Ltd. The paraffin sections of tissue were immersed in dewaxing solution, absolute ethanol, 90% ethanol and 75% ethanol for 10 minutes respectively for dewaxing. According to the instructions of anti-histone H3 (CST, 4499) antibody, the slice was put into sodium citrate repair solution and heated in microwave oven for 20 minutes under low fire for antigen repair. After incubation with 3% endogenous peroxide blocker, goat serum, respectively, for 10 minutes at room temperature, the antibodies were diluted in PBS solution according to the antibody instructions (anti-histone H3, CST, 4499, 1:200) and incubated overnight at 4°C. Then the antibodies were incubated with biotin-labeled goat anti-mouse/rabbit IgG working solution, and horseradish enzyme-labeled streptavidin ovalbumin working solution, respectively, for 10 minutes at room temperature. Finally, the films were stained and sealed with DAB staining kit (Hubei Bios Biological Technology Co., Ltd.) chromogenic solution, hematoxylin working solution, observed under a high magnification microscope, and the appropriate field of view was selected for photographic recording. The expression of histone H3 was scored by multiplying staining intensity and positive percentage. Three pathology teachers read the films in a double-blind way. The scoring rule of staining intensity was to take the average gray value of positive cells in each area as the staining intensity through Image J, and finally give four scores: High positive (3+), Positive (2+), Low Positive (1+) and Negative (0). Positive percentage: 1≤25%, 2 = 26% -- 50%, 3 = 51% -- 75%, 4≥76%. IHC staining scores ≤ 5 were defined low expression, while 6 -- 12 were high expression.

### Immunofluorescence

Cell samples were fixed with 4% paraformaldehyde. Liver tissue paraffin sections were dewaxed and placed into appropriate repair solutions for antigen repair according to antibody properties. Then the cell sample and the tissue sample were immunofluorescence stained in the same steps as below. The membrane was broken with 0.5% Triton-100 for about 15 minutes and then washed with PBS and closed with goat serum at room temperature for 30 minutes. The antibody was diluted in PBS solution according to the antibody instructions (anti-histone H3, CST, 4499, 1:400) and incubated overnight at 4°C. Then, the tissue sections were incubated with ActinGreen (KeyGEN BioTECH, KGMP001, 1:40) at room temperature for 20 minutes. Finally, the sections were incubated with secondary antibody (Cy3-AffiniPure goat anti-rabbit IgG, Wuhan Promoter Biological CO., LTD, 1:200) for 2 hours at room temperature and DAPI (Wuhan Promoter Biological Co., Ltd) for 15 minutes at room temperature before being photographed by fluorescence microscopy.

### Western blot

The RIPA buffer containing Phosphatase Inhibitor Cocktail II (MedChemExpress) and PMSF (Wuhan Promoter Biological CO., LTD) was used to split the sample on ice for 30 minutes, and then centrifuged at 12000 rpm for 15 minutes to extract the whole cell lysate. After detecting the protein concentration with BCA kit (Wuhan Servicebio Technology CO.,LTD), use 5×protein loading buffer (Wuhan Servicebio Technology CO.,LTD) to mix well, boil at 100°C for 10 minutes, and then store in the refrigerator of minus 80°C. The processed protein samples, each sample 30 μg, were added to the pre-configured 10% polyacrylamide gel after electrophoresis and transferred to PVDF (polyvinylidene difluoride) membranes. The sample was then blocked with 5% non-fat milk or 5% BSA (Albumin from bovine serum, Boster Biological Technology co.ltd) for 2 hours and incubated in diluted primary antibody working solution for 12 hours at 4°C. Finally, place in diluted secondary antibody working solution (Wuhan Promoter Biological Co., Ltd) for 2 hours at room temperature according to the antibody instructions, then use electrochemical luminescence (ECL) reagent (Yeasen Biotechnology (Shanghai) Co., Ltd.) and chemiluminescence imaging system (Bio-Rad) to exposure the western blot. Primary antibodies and working dilution ratio are listed below: anti-histone H3 (CST, 4499, 1:1000), HIF-1α (CST, 36169, 1:1000), TLR9 (CST, 13674, 1:1000), NLRP3 (CST, 13158, 1:1000), caspase-1 (Bioswamp, PAB44625, 1:2000), IL-1β (Wanleibio, WL02257, 1:2000, WL00891, 1:2000) and IL-18 (Proteintech, 02701, 1:2000). Recombinant human histone H3 protein (166242) and H3 neutralizing antibody (1791) were obtained from abcam.

### CoIP (Co-Immunoprecipitation)

The treated cellular proteins were extracted with 1% NP40, blocked using 50% Protein A/G-Agarose, added to IgG or TLR9 (CST, 13674, 1:50) rotated overnight at 4°C, and resuspended with protein loading buffer after being pulled down with 50% Protein A/G-Agarose and washed with lysate. The resulting samples were boiled at 100°C for 10 minutes and then subjected to WB analysis.

### Acquisition of cytoplasmic proteins

Extraction of cytoplasmic proteins was performed according to the instructions of the kit provided by Promoter company (QSJ-021, Wuhan Promoter Biological CO., LTD).

### SiRNA, lentivirus vector and construction of overexpression stable transgenic cell lines

The interfering RNA of TLR9 (5'-CTTCGTGGTCTTCGACAAA-3', 20uM) required for the experiments was designed and synthesized by RiboBio company (China), and the cells were transfected by Lipo3000. Lentiviral vector of H3 (CMV - 3flag - EF1 - ZsGreen - T2A - Puro) and Lv-luciferase (Lenti-luciferase-P2A-Neo) were purchased from Hanbio Biotechnology (China). After 72 hours after transfection with Lv-H3, cells were screened with purinomycin for about 14 days, and the transfection efficiency was verified by WB to establish stable histone H3 overexpressing cells. Then it was further transfected with Lv-luciferase and screened by G418 antibiotic. The transfection efficiency was verified by adding luciferase substrate *in vivo* imaging apparatus to establish stable histone H3 and luciferase overexpressing cells.

### Transfection of siRNA

The cells in good condition were inoculated in a six-hole plate with a density of about 30%. When transfection was carried out the next day, 250 μL Opti-MEMI (Gibco) and 5 μL siRNA were mixed together and incubated at room temperature for 5 minutes, 250 μL Opti-MEMI and 5 μL lipo3000 were mixed together and incubated at room temperature for 5 minutes, and then both were incubated at room temperature for 20 minutes. During this period, after the original serum culture medium in each six-hole plate was sucked, 1.5 ml of new serum free medium was added, and then 500 μL of incubated mixture was added. The solution was changed for new DMEM containing 10% FBS eight hours later, and CCK8 cell proliferation, plate cloning assay, cell migration and invasion tests were started 48 hours after transfection. The protein of cells was collected at 72 hours after transfection.

### CCK-8 (Cell Counting Kit-8) cell proliferation and plate cloning assay

The treated cells were plated on 96-well plates. There were five 96-well plates in total, which were used to detect the absorbance of 8, 24, 48, 72 and 96 hours after inoculation. The number of cells in each well was 1000, and 5 multiple wells were set for each cell. CCK8 reagent (QSJ-001, Wuhan Promoter Biological CO., LTD) was used to detect the absorbance of cells according to its operating instructions. During the detection, the old culture medium in the 96 well plate was first sucked out, then 100 μL of the mixture of fresh serum culture medium and 10 μL of CCK8 reagent were added to each well. At the same time, three blank controls were set in each 96 well plate, and 110 μL of the mixture was added, and then the absorbance was detected after being put into 37°C to react for one hour. The absorbance of 8, 24, 48, 72 and 96 hours were measured to obtain cell proliferation curves.

The cells to be tested were prepared as single cell suspensions and plated in 6-well plates at a density of 1×10^3^ cells per well, and the growth of cell clusters was observed, and when fusion between the cell clusters was imminent, the cells were fixed with 4% paraformaldehyde and stained with crystalline violet for photographs.

### Cell migration and invasion analysis

Cell migration and invasion were performed using Corning transwell polycarbonate membranes in 24-well plates (8um). 200 μl of serum-free DMEM cell suspension was inoculated in the upper chamber; 600 μl of DMEM containing 10% FBS was inoculated in the lower chamber; invasion experiments required the addition of a layer of basement membrane extract in the upper chamber. Each chamber uses 50 μl basement membrane extract, which was formed by incubating the matrix gel (Corning Matrigel Matrix) and serum-free DMEM at 37°C in a ratio of 1 to 7 for 30 minutes. After 24h or 48h of cell migration and invasion, cells that migrated into the lower chamber of the polycarbonate filter were fixed and stained with crystal violet. Cells were then observed under a microscope and photographed and counted.

### Tumour models

In the *in vivo* growth assay, MHCC-97H cells (2×10^6^) were implanted subcutaneously into the ventral side of the mice. Stable Lv-control and luciferase overexpressing MHCC-97H cells were implanted on the left side and the stable histone H3 and luciferase overexpressing MHCC-97H cells were implanted on the right side. Tumor size was calculated according to the following formula: size = (length × width)^2^/2. To monitor the size of subcutaneous tumors, mouse live imaging experiments were performed approximately 4 weeks later, and mice were executed to obtain specimens of subcutaneous tumors.

In the lung metastasis model in mice injected with tail vein, nude mice were randomly divided into two groups and injected with 2×10^6^ stable Lv-control and luciferase overexpressing MHCC-97H cells or stable histone H3 and luciferase overexpressing MHCC-97H cells, respectively. The body weights of the mice were monitored, and after approximately 4 weeks of mouse live imaging experiments were performed to observe the phenomenon of lung metastasis, and the mice were executed to obtain lung specimens from the mice.

### Statistical data analysis

Prism 5.0 (GraphPad Software, La Jolla, CA) was used for statistical analysis, and P<0.05 was considered statistically significant. The mean ± standard deviation (SD) of the data of at least three independent experiments was statistically analyzed by Student t-test or ANOVA. *P < 0.05; **P < 0.01; ***P < 0.001.

## Results

### Histone H3 is overexpressed in human HCC

**Figure 1:**
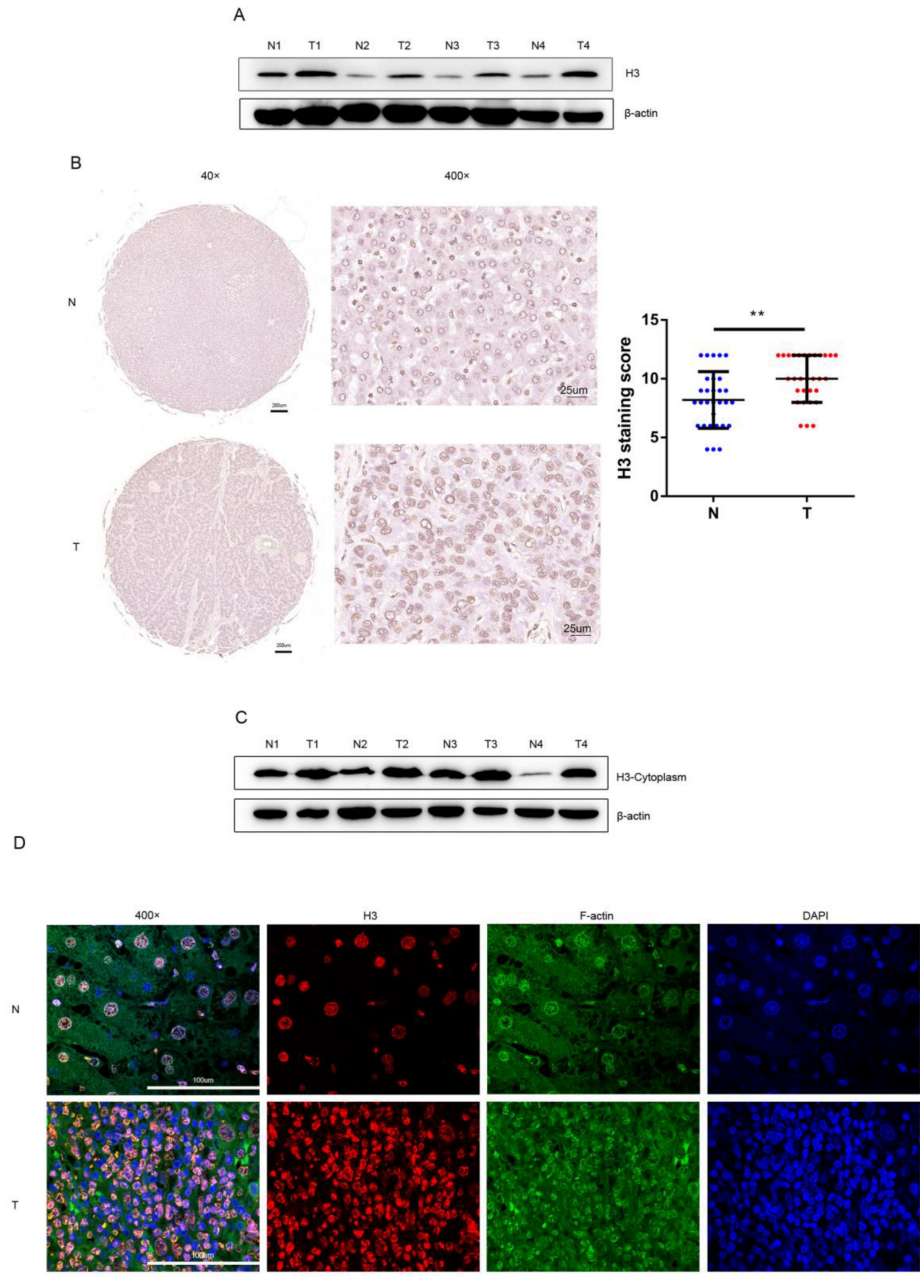
Overexpression and translocation of histone H3 in HCC (A) Histone H3 protein levels were measured with western blot in paired HCC tumor tissues (T) and adjacent non-tumor tissues (N). Protein expression results were normalized to internal control β-actin. (B) Representative images of IHC showing histone H3 expression in 30 paired HCC tumor tissues and adjacent non-tumor tissues. **P<0.01. (C) Western blot analyzed the expression of histone H3 in the cytoplasm of HCC tissues and adjacent non-tumor tissues. The expression of histone H3 in cytoplasm was measured with in HCC samples and their adjacent nontumor counterparts. (D) Immunofluorescence staining of histone H3 was performed on adjacent non-tumor tissues and HCC tissues. Red, histone H3; Green, F-actin; blue, nuclei.

After protein extraction and concentration measurement with WB (western blot) experiments were performed on the human tissue specimens, the WB results were analyzed, and we found that the level of histone H3 was higher in HCC tissues than in the adjacent non-tumor tissues (Figure 1A). While immunohistochemical staining of human HCC tissue sections was performed in 30 pairs of tissue microarray specimens from tumor and non-tumor tissues (Figure 1B), in agreement with WB results, histone H3 in HCC tissues stained darker than in adjacent non-tumor tissues, and the staining score of histone H3 in HCC tissues was relatively higher and statistically significant. This suggests that we have overexpression of histone H3 in clinical HCC patients. In addition, we found higher levels of cytoplasmic histone H3 in HCC tissues by extracting cytoplasmic proteins from tumor and adjacent non-tumor tissues with WB experiments (Figure 1C). Also, in the immunofluorescence of Figure 1D, we found that in non-tumor tissues, the staining of histone H3 completely overlapped with the nuclear staining of DAPI, which was retained intact in the nucleus in the normal state. In contrast, in HCC tissues, we could see that histone H3 staining was missing in the nucleus, i.e., it did not overlap with DAPI, and the red stain histone H3 overlapped the green stain F-actin of the cytoplasm to yellow, which meant that histone H3 was visible in the cycloplasm. This suggests that there is a translocation of histone H3 from the nucleus to the cytoplasm in HCC.

### Hypoxia leads to the translocation of histone H3 from the nucleus to the cytoplasm in HCC cells

**Figure 2:**
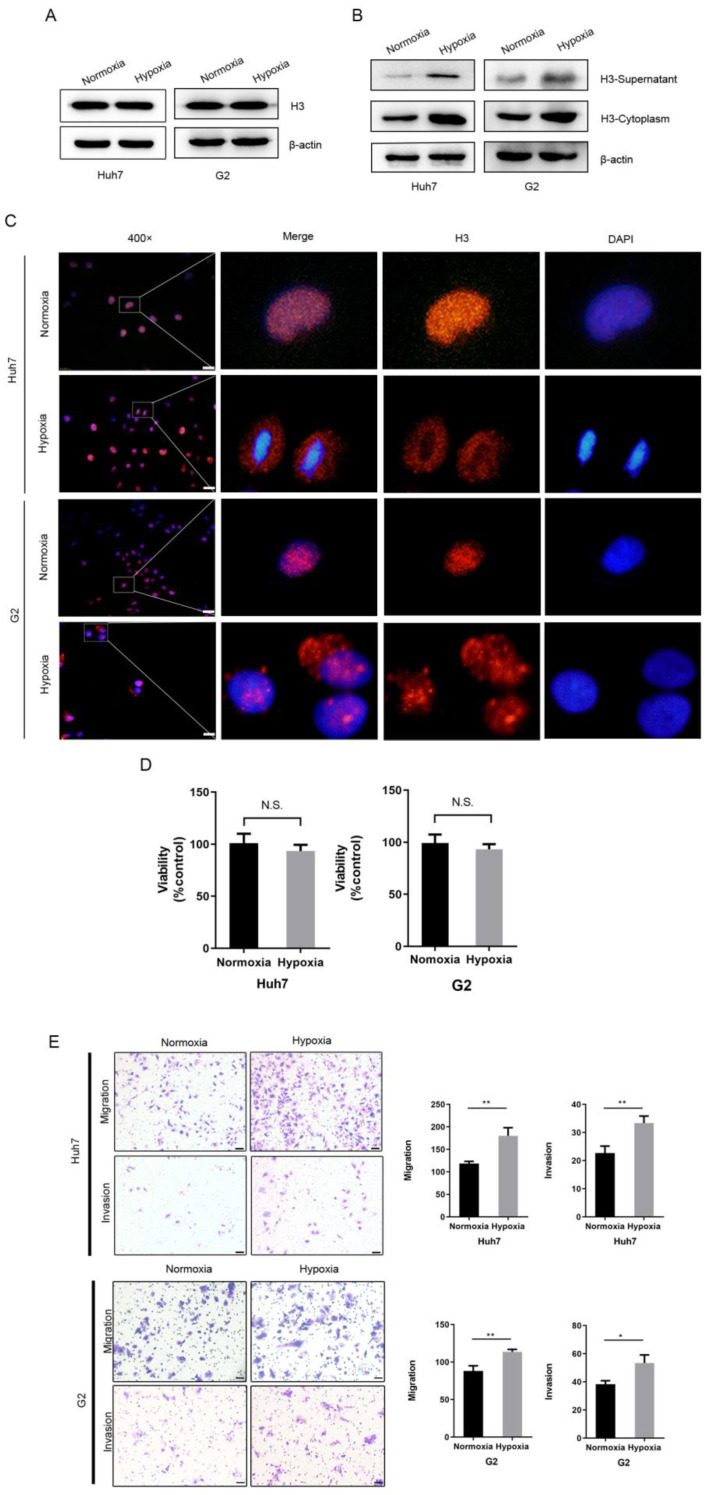
Hypoxia leads to the translocation of histone H3 from nucleus to cytoplasm in HCC cells and promotes HCC cells metastasis (A-B) Western blot analysis the expression of histone H3 in whole cell protein, cytoplasmic protein and supernatant of Huh7 and G2 cells after normoxic and hypoxic (1% O_2_) culture. (C) Huh7 and G2 were stained by immunostaining after normoxia and hypoxia. Red, histone H3; blue, nuclei. Scale bar = 25 µm. (D) The viability of Huh7 and G2 cells under normoxia and hypoxia for 24h was compared by CCK-8 assay. *P<0.05, **P<0.01, N.S. no significance. (E) Transwell migration and invasion studies were performed for 24h under normoxia and hypoxia. The numbers of migratory and invasive cells were quantified in normoxia and hypoxia of Huh7 and G2 cells. Magnification = 200×, scale bar = 50 µm.

Since hypoxia is very common in HCC as a solid tumor cell, we hypothesized that it is hypoxia that can cause the translocation of histone H3 from the nucleus to the cytoplasm. To investigate the role of the hypoxic microenvironment in histone H3 carcinogenesis in HCC, we treated HCC cells using a physical hypoxia method, culturing Huh7 and G2 cells under normoxia (21% O_2_) or hypoxia (1% O_2_) conditions, respectively. After exposure to hypoxia, we collected the cells and analyzed the whole cell lysate, cytoplasmic proteins or supernatant proteins before and after hypoxia, and by WB experiments we found an increase in histone H3 in the cytoplasm and supernatant, while there was no significant change in whole cell proteins (Figure 2A-B). Also, similar to the immunofluorescence results of HCC tissues, histone H3 in Huh7 and G2 cells *in vitro* was located in the nucleus in overlap with the staining of DAPI during normoxia, whereas after hypoxia the staining was absent in the nucleus but was evident in the cytoplasm (Fig. 2C), indicating this translocation of histone H3. Thus, these results suggest that hypoxia causes a translocation of histone H3 from the nucleus to the cytoplasm in HCC cells. To further confirm the role of hypoxia in the biological behavior of HCC cells, we examined the cellular activity and the ability of cells to migrate and invade. In Figure 2D, we assessed the cell viability of HCC cells by using the CCK-8 cytometric assay, and by examining the statistical analysis of the absorbance comparison of HCC cells cultured under hypoxic and normoxic conditions, we found no substantial change in cell viability under hypoxia and normoxia. However, corning transwell polycarbonate membranes chamber experiments by Figure 2E showed that the migration and invasion ability of Huh7 cells increased under hypoxic culture for 24 hours. These findings demonstrate that hypoxia promotes the migration of HCC cells.

### Hypoxia and histone H3 activate NLRP3 inflammasome through TLR9

**Figure 3:**
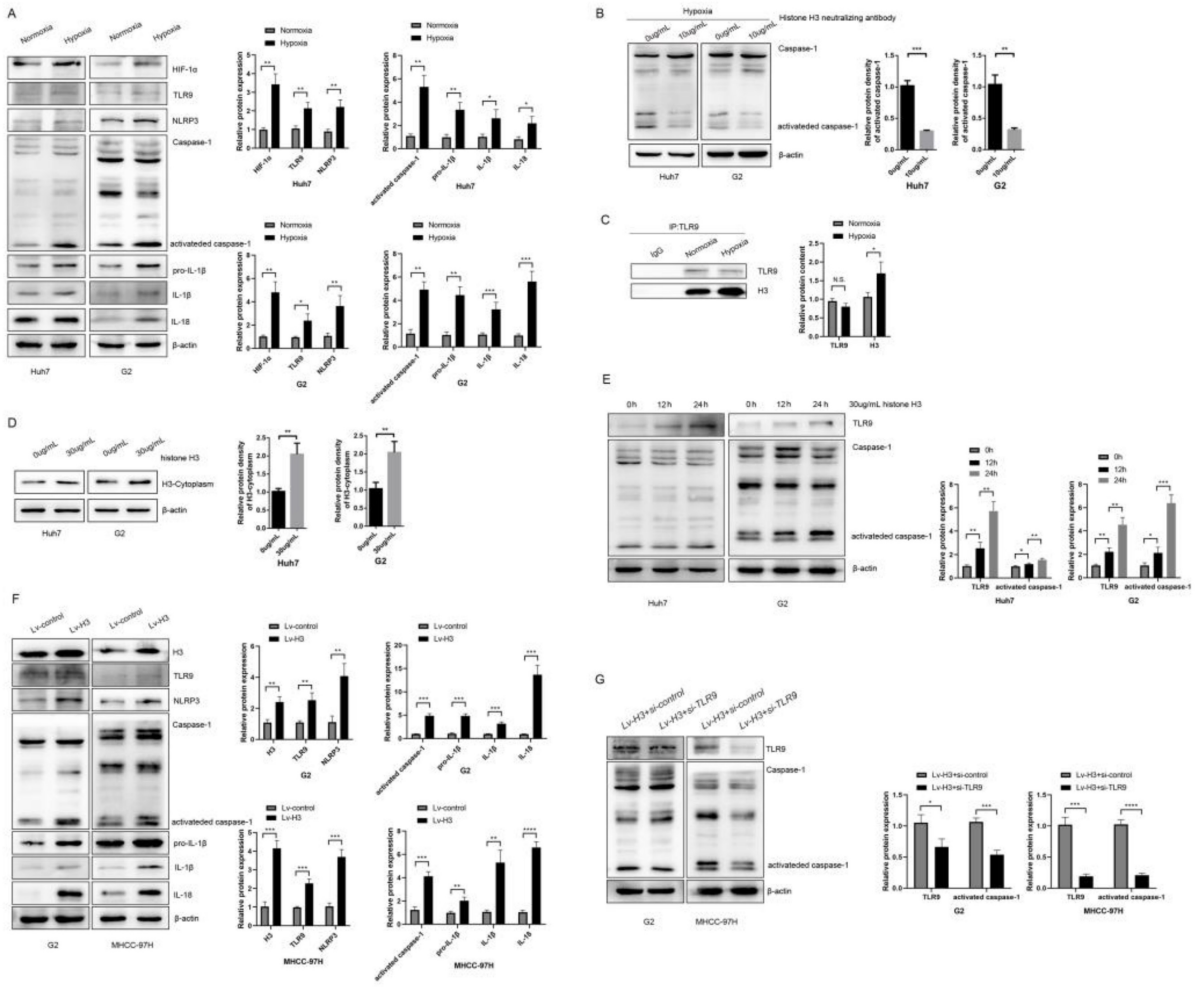
Hypoxia and histone H3 activate NLRP3 inflammasome through TLR9 (A) The expression of HIF-1α, TLR9, NLRP3, Caspase-1, IL-1β and IL-18 were determined by western blot in Huh7 and G2 cells exposed to 24h hypoxia. *P<0.05, **P<0.01, ***P<0.001, ****P<0.0001, N.S. no significance. (B) The expression of caspase-1 in Huh7 and G2 cells treated with 10 μg/ml anti-Histone H3 neutralizing antibody under hypoxia. (C) The IP results showed that the content of histone H3 bound by TLR9 increased after hypoxia. (D) The content of H3 in cytoplasm of Huh7 and G2 cells treated with 30 μg/ml recombinant human histone H3 for 24 hours. (E) The expression of caspase-1 in Huh7 and G2 cells treated with 30 μg/ml recombinant human histone H3 for diverse times. (F) The expression of TLR9, NLRP3, caspase-1, IL-1β and IL-18 were significantly increased in stable histones H3-expressing cells via western blot analysis. (G) Western blot analysis for caspase-1 from stable histones H3-expressing cells after transfected with TLR9 siRNA. Lv-control+si-control group was stable Lv-control HCC cell to be transfected with si-control; Lv-H3+si-TLR9 group was stable histones H3-expressing cells to be transfected with si-TLR9.

NLRP3 is an important player in causing inflammation and can be activated by multiple stimuli leading to caspase-1 activation, as well as maturation and secretion of substrates such as IL-1β and IL-18. After hypoxia, we collected whole cell lysates and found that the expression of HIF-1α and TLR9 was increased in both HCC cell lines after hypoxia (Figure 3A). And we found that activated caspase-1, IL-1β and IL-18 were all upregulated in Huh7 and G2 cells exposed to hypoxia (Figure 3A). In order to verify whether the release of H3 under hypoxia can cause the activation of caspase-1, we used the neutralizing antibody of histone H3 while treating Huh7 and G2 cells under hypoxia. The results showed that the activation of caspase-1 decreased after neutralizing the effect of histone H3 (Figure 3B). Meanwhile, the results according to CoIP (Figure 3C) showed that the level of histone H3 bound to TLR9 was increased after hypoxia. To investigate whether histone H3 could activate caspase-1, recombinant human histone H3 protein was treated onto HCC cells under normoxic cell culture. By extracting the cytoplasmic protein of cells, it could be seen that the content of H3 in the cytoplasm increased after the treatment of recombinant human histone H3 (Figure 3D).

Similar to Huang et al^13^, treatment with recombinant histone H3 (30ug/ml) promoted caspase-1 activation in HCC cell lines over time (Figure 3E). To further investigate the specific mechanism of action of histone H3 on HCC cells, we collected whole cell lysates from stable histone H3 overexpressing cells and control cells. WB analysis showed that the levels of TLR9, NLRP3, activated caspase-1, IL-1β and IL-18 were upregulated in stable histone H3 overexpressing cells (Figure 3F). To investigate whether histone H3-induced caspase-1 activation was TLR9-dependent, we treated stable histone H3-expressing cells with TLR9 siRNA and collected whole cell lysates. We found that the protein cleavage level of caspase-1 was reduced by WB assay after TLR9 siRNA treatment (Figure 3G). Thus, in HCC cells, hypoxia and histone H3 could cause upregulation of NLRP3 inflammasome via TLR9, while the upregulation of inflammasome was suppressed after TLR9 inhibition. Taken together, these results confirm that hypoxia and histone H3 can activate NLRP3 inflammasome.

### Histone H3 promotes HCC cell proliferation and metastasis via TLR9 *in vitro*

**Figure 4:**
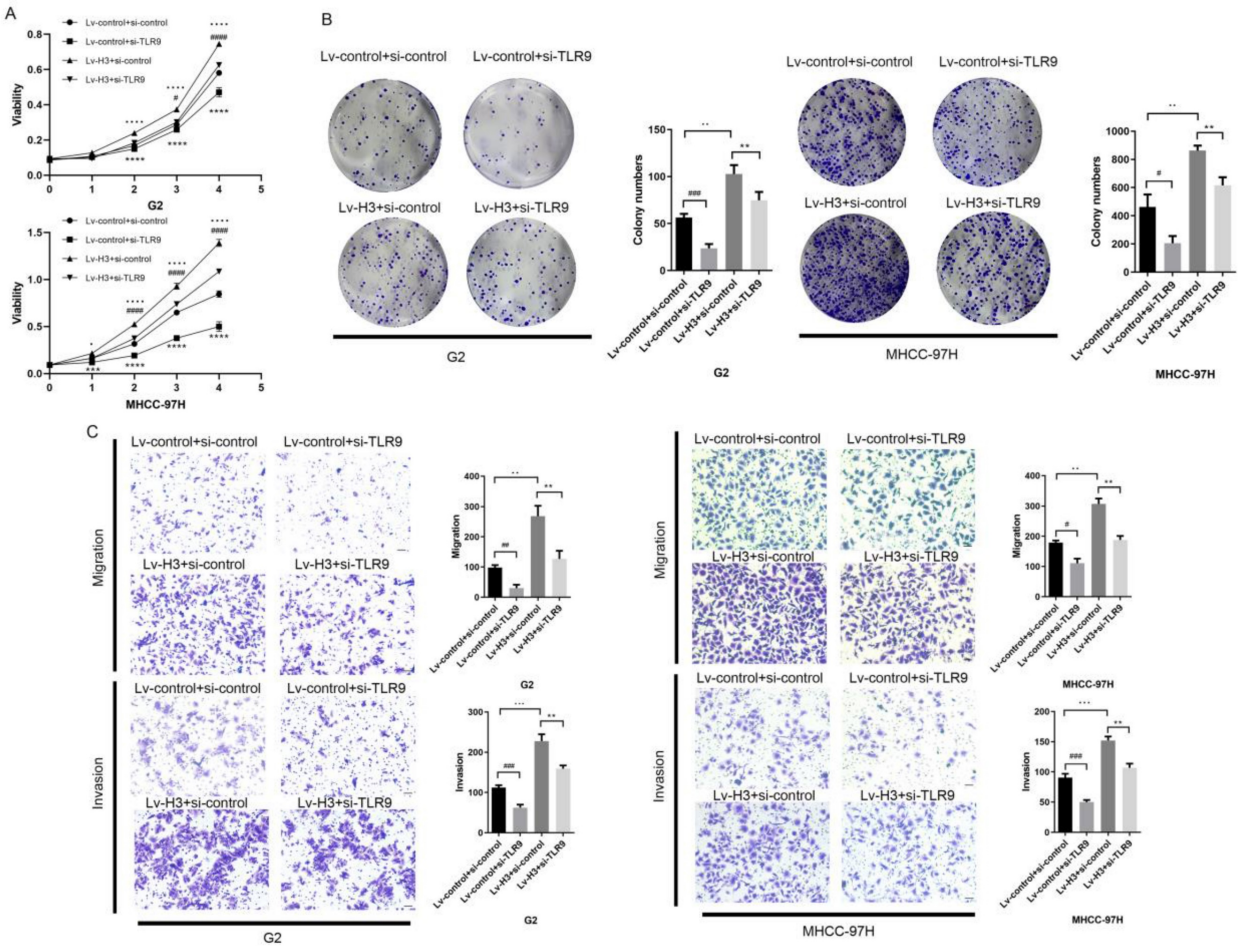
Histones H3 promotes HCC cells proliferation and metastasis through TLR9 *in vitro* (A) CCK-8 assay was performed to determine the proliferation of G2 and MHCC-97H cells with various treatments. *P<0.05, **P<0.01, ***P<0.001, ****P<0.0001. # represents the comparison and analysis between Lv-control+si-control and Lv-control+si-TLR9; represents the comparison and analysis between Lv-control+si-control and Lv-H3+si-control; * represents the comparison and analysis between Lv-H3+si-control and Lv-H3+si-TLR9. (B) Colony-forming assay was performed to determine the proliferation of G2 and MHCC-97H cells with various treatments. (C) G2 and MHCC-97H cells with various treatments were seeded to migrate or invade for 24h or 48h. The number of migratory and invasive cells was quantified. Magnification = 200×, scale bar = 50 µm.

To re-validate the role of histone H3 and TLR9 in HCC cells, we performed CCK-8 cell proliferation and colony formation assays. From the results (Figure 4A-B), it was shown that the proliferation and colony formation of HCC cells were enhanced after overexpression of histone H3 with statistical study analysis. After further re-inhibition with TLR9 siRNA, their ability decreased. Based on the migration assay in Figure 4C and the invasion assay in Figure 4D, we found that the migration and invasion ability of stable histone H3 overexpressing cells was significantly enhanced compared with the control group. Similar to the results of the proliferation assay, the migration and invasion of stable histone H3 overexpressing cells were reduced after TLR9 siRNA treatment (Figure 4C-D). However, the effect of H3 on the proliferation and metastasis of HCC cells could not be completely inhibited by the knockdown of TLR9, which suggested that histone H3 might also cause other molecules or pathways to play a role. In the statistical analysis of the above studies, the P values for the corresponding changes were less than 0.05, all of which were statistically significant. These findings confirm that histone H3 promotes the proliferation and metastasis of HCC cells via TLR9 *in vitro*.

### Histone H3 promotes HCC cells proliferation and metastasis *in vivo*

**Figure 5:**
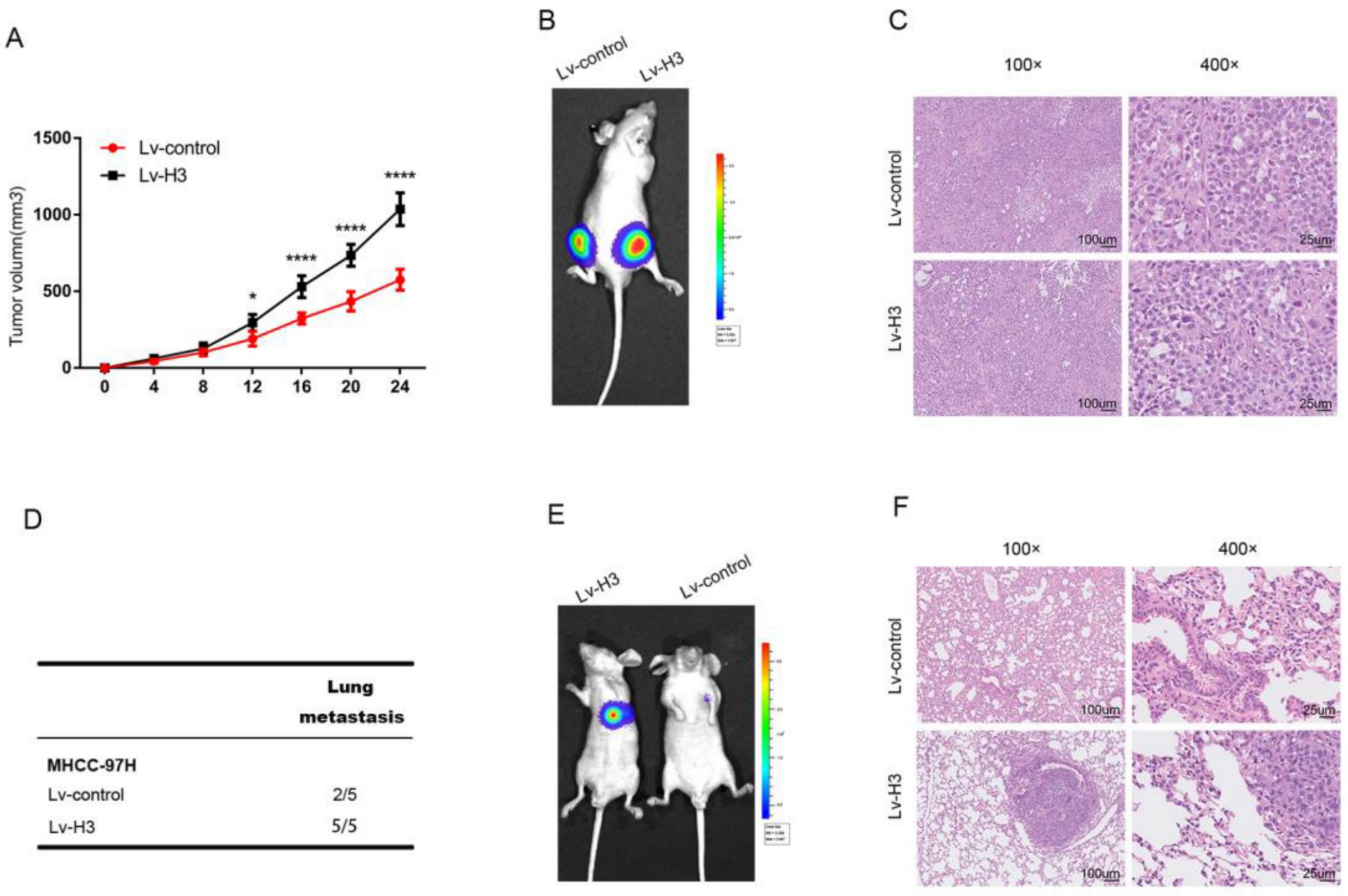
Histones H3 promotes HCC cells proliferation and metastasis in vivo Tumor growth curves (A) and representative H&E staining (B) shown after stable histones H3-expressing cells were engrafted in flanks of BALB/c nude mice. *P<0.05, ****P<0.0001. (C) showed luciferase signal of control cells (the left flank) and stable histones H3-expressing cells (the right flank) in one BALB/c nude mice. Lung metastasis experiments were performed by tail vein injection model. Statistical table of pulmonary metastasis (D), representative luciferase (E) signal images and H&E staining of lung tissues (F) were presented.

To determine the specific role of histone H3 in HCC, we transfected MHCC-97H cells with lentivirus to establish stable histone H3 and luciferase overexpressing cells, which were transplanted to both sides of BALB/c nude mice. Lv-control cells were grown on the left side, and Lv-H3 overexpressing cells were grown on the right side of the same mice. Comparison of the volume growth curves of the subcutaneous tumors in Figure 5A, and the live imaging results of representative mice in Figure 5B show that the Lv-H3 overexpressing tumor nodules were larger in volume compared with the Lv-control cells. H&E (Hematoxylin and eosin) results were shown in Figure 5C, where the nuclei of Lv-H3 overexpressing tumor nodules were relatively larger with darker staining. To determine whether histone H3 was involved in the metastasis of HCC, we used a mouse tail vein lung metastasis model. Stable histone H3 and luciferase overexpressing MHCC-97H cells were injected into mice through the tail vein. Four weeks after injection, mice were examined for lung metastasis by live imaging and executed. The results were shown by Figure 5D that lung metastasis occurred in two of the five control mice and in all of the five Lv-H3 overexpressing group mice. It indicated that the incidence of lung metastasis was higher in the Lv-H3 overexpressing group than the control group. The live imaging results from representative mice in Figure 5E, showed that the bioluminescence signal in the lungs of mice in the Lv-H3 overexpressing group was significantly stronger than that in the control group. The H&E staining of specimens with lung metastases was shown in Figure 5F, we could see that mouse in the Lv-H3 overexpressing group had obvious intrapulmonary metastasis. Taken together, the experimental results confirm that histone H3 promotes the proliferation and metastasis of HCC cells *in vivo*.

## Discussion

A complex pathological process accompanies the development of HCC^14^, in which hypoxia and associated oxidative stress are common pathophysiological factors that occur after various risk factors that cause infection and inflammation^15^. Under the combined influence of multiple conditions such as chronic hypoxia and chronic inflammation, cells undergo alterations in epigenetic or genetic properties that exacerbate the malignancy of tumor cells and tumor progression^16^. Meanwhile, HCC is mainly treated by surgical resection, liver transplantation, chemotherapy and radiotherapy. However, during treatment, states such as hypoxia-ischemia also always occur and can promote the deterioration of HCC cells in several ways, which can seriously affect the outcome^17^. However, the hypoxia-mediated mechanism of HCC remains unclear and more research is needed to elucidate in order to control the development of HCC and improve the treatment outcome^14^.

HCC is most often associated with inflammation of the liver^18^, and inflammation occurs in almost all patients with HCC, and the main signaling pathway leading to acute and chronic inflammation is through the activation of inflammasomes^6^. In contrast, the best known inflammasome by far is NLRP3, an intracellular sensor composed of multiple proteins in the cell, mainly consisting of the dot protein ASC and pro-caspase-1^19^. NLRP3 acts as a protein complex scaffold that is linked by ASC to the effector protein pro-caspase-1. When NLRP3 is activated, it leads to pro-caspase-1 activation and subsequent cleavage of IL-1β and IL-18 precursors into their active forms^20^. Caspase-1 is activated in this process as an inflammatory caspase in response to pathogens and endogenous mediators^21^. In addition to attracting and activating immune cells and inducing inflammation, caspase-1 also promotes inflammation by promoting cell survival and metastasis to influence tumor progression^22^. Studies have shown that although the role of inflammasome in cancer is unclear still needs to be studied in depth, it is clear that abnormal NLRP3 inflammasome activation is associated with tumorigenesis. NLRP3 inflammasome inhibit tumorigenesis in colon cancer, while promoting tumor growth, proliferation, invasion and metastasis in lung, breast and other tumors^5^. In this study, our experimental results revealed that histone H3 promotes NLRP3 inflammasome activation, which leads to proliferation, metastasis and invasion of HCC.

HCC is the cumulative result of genomic changes and epigenetic modifications in somatic cells, with a great deal of molecular heterogeneity. An important hallmark of aging is changes in chromatin structure, a change that may be caused by reduced levels of histones, while some studies have shown that overexpression of histones can mitigate these changes and contribute to lifespan extension^23^. At the same time, histones damage endothelial cells, activate the coagulation system, cause the release of a series of corresponding cellular inflammatory factors, activate TLRs on the cell surface and lead to immune dysfunction, platelet aggregation, thrombosis, and damage to various organs of the body^24^. This suggests to us that the overexpression levels of histones themselves can cause an increase in cellular viability and the development of inflammation in the body. Meanwhile, a recent study by Huang et al^13^ showed that endogenous histones act as a molecular damage model after ischemic injury and induce inflammation through TLR9 signaling. Previously, we reported that hypoxic HCC cells undergo caspase-1 activation, which is dependent on the release of extracellular HMGB1 and subsequent activation of TLR4 and RAGE signaling pathways^12^. HMGB1 is a DNA-binding protein that is translocated into the cytoplasm upon cellular stress and is actively or passively released outside the cell depending on the cell type and stressor^12^. Here, we found that in HCC cells under hypoxia, histone H3 has a similar phenomenon to HMGB1. We hypothesize that persistent hypoxia in HCC leads to cellular release of histones into the intracellular and extracellular compartments, triggering inflammation via self or surrounding cells. However, it is unclear whether extracellular histone H3 is passively released from dead cells or actively secreted from damaged living cells.

TLRs are highly conserved proteins that interact with PAMPs (pathogen-associated molecular pattern molecules) and DAMPs (damage-associated molecular pattern molecules) to trigger exogenous and endogenous inflammatory responses^25^. As a member of the TLRs family, TLR9 has received increasing attention for its role in HCC^26^. TLR9 can usually be activated by microbial DNA sequences containing unmethylated CpG nucleotides^27^. In previous studies, we found that TLR9 was overexpressed in human HCC tissues where hypoxia was common and in HCC cells exposed to hypoxia, and that TLR9 was required for HCC cell proliferation under hypoxic conditions. Furthermore, upon hypoxia in HCC cells, nuclear HMGB1 translocates to the cytoplasm and binds to free mtDNA, which further activates TLR9^28^. Since HMBG1 itself can bind to receptors called RAGE and cause inflammatory signals, and TLR9 can bind to and be activated by CpG oligodeoxynucleotides, enhances the inflammatory response when used in combination with HMGB1 by activation of RAGE^29^. In the present experiment, we found an increase in histone H3 bound to TLR9 after hypoxia by CoIP experiments. Similar to our study, Huang et al. found that histone-TLR9 activation mediates the production of mitochondrial ROS and consequently activates the NLRP3 inflammasome. This leads us to speculate that the activation of TLR9 by histone H3 may be due to DNA-TLR9-dependent DAMP action generated by histone H3 and free mtDNA. However, the effect of H3 on the proliferation and metastasis of HCC cell could not be completely inhibited by the knockdown of TLR9, which suggests that histone H3 might also cause other molecules or pathways to play a role. It has been shown that histone H3 could regulate the activation of inflammasome through the expression of NLRP3 mediated by V-set and immunoglobulin domain containing 4 (VSIG4)30. The mechanistic of histone H3 and the relationship between histone H3 and TLR9 in HCC still need to be further explored.

## Conclusions

Conclusively, hypoxia may play a key regulatory role for histone H3 in HCC invasion and metastasis, and the release of histone H3 from the nucleus to the cytoplasm and extracellular in hypoxic conditions promoted the proliferation and metastasis of HCC. In addition, through *in vitro* experiments such as CCK8, plate cloning, corning transwell polycarbonate membranes chamber experiments and *in vivo* mouse subcutaneous tumor and tail vein lung metastasis models, we found that histone H3 was closely associated with tumor proliferation and metastasis. And in this process, histone H3 activated caspase-1 through TLR9, while the proliferation and metastatic ability of stable histone H3 overexpressing cells after inhibition of TLR9 were all decreased. Taken together, these findings suggested a role for protein changes in histone H3 itself in HCC, opening up new perspectives on the role of histones in tumors and opening up new prospects for what can be a potential HCC treatment.

## Supplementary Material

Supplementary table 1.

## Abbreviations

HCC: hepatocellular carcinoma
ROS: reactive oxygen species
HMGB1: high mobility group protein 1
TLR4: toll-like receptor 4
TLR9: toll-like receptor 9
RAGE: receptor for advanced glycation endproducts
DMEM: dulbecco's modified eagle medium
FBS: fetal bovine serum
PVDF: polyvinylidene difluoride
CoIP: co-immunoprecipitation
CCK-8: cell counting kit-8
WB: western blot
H&E: hematoxylin and eosin
PAMPs: pathogen-associated molecular pattern molecules
DAMPs: damage-associated molecular pattern molecules
